# Differential gene expression between the vigorous and dwarf litchi cultivars based on RNA-Seq transcriptome analysis

**DOI:** 10.1371/journal.pone.0208771

**Published:** 2018-12-12

**Authors:** Fuchu Hu, Zhe Chen, Jietang Zhao, Xianghe Wang, Wenbing Su, Yonghua Qin, Guibing Hu

**Affiliations:** 1 State Key Laboratory for Conservation and Utilization of Subtropical Agro-bioresources/Key Laboratory of Biology and Genetic Improvement of Horticultural Crops (South China) in Ministry of Agriculture, College of Horticulture, South China Agricultural University, Guangzhou, China; 2 Key Laboratory of Tropical Fruit Tree Biology of Hainan Province/Institute of Tropical Fruit Trees, Hainan Academy of Agricultural Science, Haikou, China; Key Laboratory of Horticultural Plant Biology (MOE), CHINA

## Abstract

Litchi (*Litchi chinesis* Sonn.) is the most economically significant member of *Sapindaceae* family, especially in sub-tropical regions. However, its tall tree body often brings many inconveniences to production management. In order to modify the tree size or growth for productivity optimization and simplifying management, it is urgent to reveal the dwarf mechanism of litchi for dwarfing rootstocks or cultivar breeding. However, to date, the mechanisms on litchi dwarfism is still poor known. In the present study, transcriptome profiling were performed on *L*. *chinensis* cv. ‘Feizixiao’ (FZX, vigorous cultivar) and ‘Ziniangxi’ (ZNX, dwarf cultivar). A total of 55,810 unigenes were obtained, and 9,190 unigenes were differentially expressed between vigorous and dwarf litchi samples. Gene functional enrichment analysis indicated that the differentially expressed unigenes (DEGs) were related to phytohormone metabolism and signal transduction, and energy metabolism pathways. In particular, *GA2ox* were only up-regulated in ZNX samples, indicating GA might play an important role in regulating huge difference between vigorous and dwarf litchi cultivars. In addition, the *35S*::*LcGA2ox* transgenic tobacco plants were dwarf and had smaller leaves or branches than wild type plants. Our study provided a series of candidate genes to reveal the mechanism of litchi dwarf.

## Introduction

In fruit production, tree architecture requires unique horticultural practices, including grafting, pruning, and training [1]. These practices need to be designed to maximize productivity for a minimum of expense. Due to the high cost of labor, especially in developed countries, the modifying of tree size or growth is critical for productivity optimization and simplifying management [2]. Dwarfing rootstocks and/or interstocks have been available and widely used for fruit and nut trees to create orchards with smaller and easier-to-handle trees [3]. For example, the use of dwarfing rootstocks has become very common in important temperate fruit trees like apple, pear, peach, and cherry [4–7]. Unfortunately, in most tropical and subtropical fruit trees (i.e., litchi, longan), dwarfing rootstocks are not commercially available. Furthermore, the mechanism how rootstocks dwarf fruit trees is not clear [8]. Evergreen subtropical crops such as litchi and mango are often hedged or pruned to control the size of the trees [9]. In order to control tree size, plant growth regulators are also applied [10, 11], but it will increase the production cost. Genetic engineering offers a promising approach for developing dwarfing fruit trees to minimize negative efforts.

Thus far, the progress on genetic manipulation of tree size has been comparatively limited. The barriers include the large size and the long generation times [12]. Quantitative trait locus (QTL) analysis has been performed to aid the tree size breeding [13, 14]. However, QTLs have limitations as molecular markers for the early breeding selection [12]. Therefore, the identification and functional analysis of genes associated with tree size is critical for both conventional breeding and genetic engineering. Fruit size is assumed to be controlled by polygenes and the molecular mechanism is not fully understood [15]. Dwarf mutants are effectively used in identification of dwarfism related genes in many fruit trees. Chen et al. [16] obtained a dwarf mutant of ‘Williams’ variety of banana and elucidated that GA might play a pivotal role in its dwarfism. Recently, the brachytic dwarfism trait (*dw*) of peach trees was found due to a nonsense mutation in the gibberellic acid (GA) receptor *PpeGID1c* [1].

GAs play fundamental functions in plant growth and reducing level of active GAs causes the dwarf phenotype in plants [17]. Therefore, the attempts to alter GAs metabolism and/or signaling have been performed to control plant size [18, 19]. GA 20-oxidase (GA20ox), GA 3-oxidase (GA3ox), and GA 2-oxidase (GA2ox) catalyzing later reactions are key enzymes controlling GA biosynthesis [20]. These enzymes are encoded by multigene families having different temporal and spatial expression patterns [21]. Down-regulation of *GA20ox* and *GA3ox* resulted in decreasing GA levels and displayed a dwarf phenotype. For instance, the suppressed expression of *GA20ox* gene in apple caused dwarfism, which was restored by the application of GA_3_ [22]. By contrast, overexpression of *GA2ox* genes, which encode enzymes converting active forms of GAs to inactive forms, also produces dwarf plants [23]. Likewise, overexpression of the *DELLA* genes, which act to repress GA signaling, leads to dwarfism in apple [24]. In addition to GA, brassinosteroids (BRs) have also been linked to dwarfism [12]. Additionally, crosstalk between GA and other phytohormones (i.e. auxin, BRs, ethylene) play essential roles in plant height control [19].

Litchi (*Litchi chinesis* Sonn.) is the most economically significant member of Sapindaceae family [25]. It has a very long history in China and is famous for its red skin and juicy sweet aril. The litchi tree is medium to large, which can grow up to 10–12 m or even 20 m [26]. Traditionally, litchi are planted with wide spacing with about 70–80 trees per hectare. Such plantings waste land resource in the early years. What is more, there are problems with harvesting, spraying and protection from birds and bats for these large trees [27]. Another problem is, with V-shaped branches, litchi shoots are easily broken off by strong winds [28]. Therefore, conventional high litchi tree architecture costs vast labor and capital on orchard management, and it is extremely necessary for growers to carry out dense and dwarfing planting to reduce production cost. In our previous study, we found litchi cultivars ‘Ziniangxi’ and ‘Ya1’ are two of the rare potential dwarf litchi germplasms [29]. And the biological and anatomic characteristics of stem of ‘Ziniangxi’ are consistent with what Tombesi et al. [30] and Zorić et al. [31] had reported on peach and cherry, respectively. In this case, ‘Ziniangxi’ litchi might be an excellent germplasm to develop dwarfing and dense plantation on litchi.

In this study, we chose two litchi cultivars ‘Feizixiao’ and ‘Ziniangxi’ with different vigorous levels and sequenced the leaves and apical buds by RNA-seq. By comparing anatomical and differential gene expression in vigorous and dwarf cultivars, we hypothesize that genes related to phytohormones pathways and energy metabolism pathways might play important roles in litchi dwarfism.

## Materials and methods

### Plant materials

*Litchi chinensis* cv. ‘Feizixiao’ (FZX) and ‘Ziniangxi’ (ZNX) were planted in the innovation experimental orchard of Hainan Academy of Agricultural Science, Haikou, China. FZX is a vigorous cultivar, with long, sparse, fragile branches, and large, narrow, deep glossy green leaflets. ZNX is a dwarf cultivar, with thin and open spreading branches. Mature leaves and apical buds were collected from the mature trees of FZX and ZNX, respectively. All these samples with three biological replicates were harvested at the same time to avoid the different transcript due to circadian rhythm factors. Trees used in the experiment were not chemically treated or pruned. All samples were immersed in liquid nitrogen and stored at -80°C for RNA extraction.

### Paraffin section microscopy

Paraffin section microscopy was performed following the protocols described by Chen et al. [32]. The paraffin sections were observed using the photomicroscope.

### RNA extraction, cDNA library construction, and sequencing

Total RNA was isolated using the Quick RNA Isolation Kit and treated with DNase I (TaKaRa, Japan) to remove genomic DNA contamination. RNA integrity was assessed using the RNA Nano 6000 Assay Kit of the Bioanalyzer 2100 system (Agilent Technologies, CA, USA). The mRNA enrichment, mRNA fragmentation, second-strand cDNA synthesis, size selection, PCR amplification and sequencing using an Illumina HiSeq (San Diego, CA, USA) were performed at the Novogene Institute (Novogene, Beijing, China).

### Data filtering, *de novo* assembly and functional annotation

Raw data (raw reads) in fastq format were trimmed and filtered using Trimmomatic v0.33 [33]. High-quality reads were obtained by removing reads containing adapters, reads containing poly-N and low-quality reads. At the same time, the Q20, Q30 and GC content were calculated. High-quality reads were aligned to the SSU and LSU rRNA sequences download form silva database [34] using bwa [35]. Next, rRNA reads were removed by a home-made perl script and clean data were obtained. Reads from all libraries were *de novo* assembled using Trinity [36] and CD-hit [37] into a gene set that served as the reference for subsequent analysis.

Gene function was annotated based on the highest similarity in the following databases: Nr (http://www.ncbi.nlm.nih.gov, NCBI non-redundant protein sequences, with a cut-off e-value of 1e^-5^), COG (http://www.ncbi.nlm.nih.gov/COG, Clusters of Orthologous Groups of proteins), KO (http://www.genome.jp/kegg, KEGG Orthology database). GO (Gene Ontology) functional annotation was performed using Blast2GO (v2.5.0) software.

### Differential expression analysis and functional enrichment analysis

Gene expression levels were calculated based on the length of the gene and reads count mapped to this gene using the FPKM (fragments per kilobase of transcript sequence per millions base pairs) method [38]. Differential expression analysis for each sequenced library was performed using DESeq [39]. The *P* values were adjusted using the Benjamini & Hochberg method [40]. The corrected *P* value of 0.05 and abs |log2(Fold change)| of 1 were set as the threshold for significantly differential expression. GO and KEGG pathway enrichment analysis were performed by TBtools (http://cj-chen.github.io/tbtools). REVIGO was applied to visualize the GO enrichment results [41].

### Quantitative real-time PCR analysis

Total RNA was isolated as described above and reverse transcribed with oligo (dT)_18_ primers using M-MLV reverse transcriptase (Invitrogen, USA). Transcript levels were analyzed using quantitative RT-PCR with the DyNAmo Flash SYBR Green qPCR kit (Thermo, USA) and the CFX96 qPCR System (Bio-Rad, USA). Gene-specific primers were designed using the Primer 5.0 program (PREMIER Biosoft International, Canada) and listed in S1 Table. All reactions were performed in triplicate with three biological replicates. All reactions were normalized using the Ct values corresponding to *Lcactin* gene (HQ615689). Unigene expression levels were calculated using the 2^ΔΔCT^ method [42].

### Isolation of *LcGA2ox* genes and functional analysis in tobacco

The full length of *LcGA2ox1-3* genes were amplified by PCR with primers (S1 Table) through high fidelity PCR (Prime STARTM HS DNA polymerase, Takara). The PCR products were digested with *Bam*HI and *Sac*I respectively, and fused into the plant binary vector pBI121 digested by the same enzymes. The generated binary vectors were transferred into *Agrobacterium tumefaciens* strain LBA4404 using the freeze-thaw method. Transformation of *Nicotiana tabacum* was performed using the leaf disc method as previously described by Chen et al. [43]. Transgenic tobacco plants were confirmed by PCR using genomic DNA. The transgenic (T1) tobacco plants were selected and used for morphological analyses. For morphological analysis, plant height, stem diameter, leaf size, internode number and length were measured.

### Statistical analysis

Statistical analyses were performed with SPSS software (SPSS, Chicago, IL). One-way analysis of variance (ANOVA) was used to evaluate the difference on each sample. Heatmap diagram were analyzed using R software with pheatmap methods. Significant correlations between qRT-PCR and transcriptome data were analyzed with SPSS software using Pearson’s correlation as the statistical metric. Significant correlations were considered only when an adjusted P value was lower than 0.05.

## Results

### Anatomical observation

**Fig 1:**
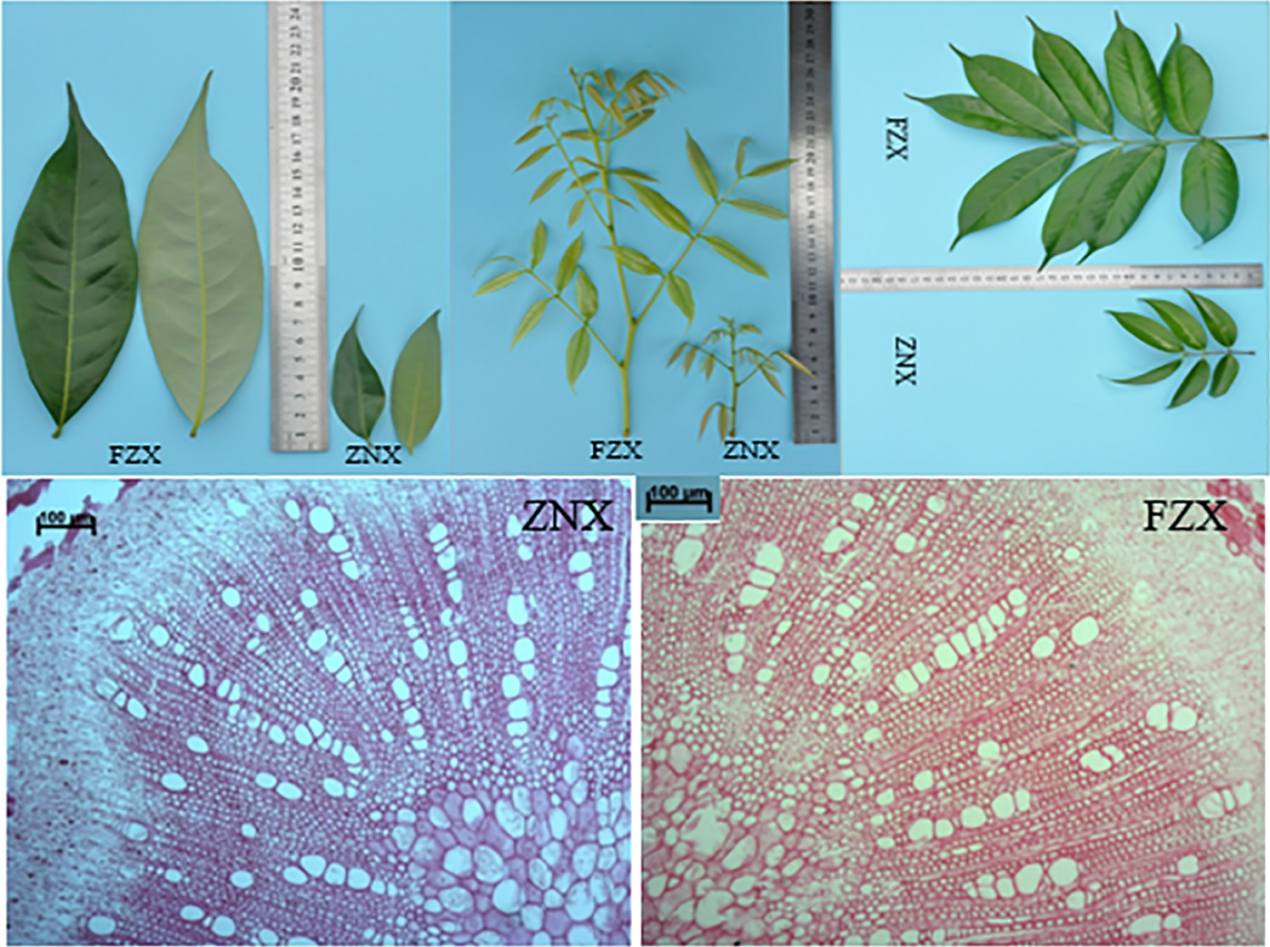
The comparison of leaf, compound leaf, terminal bud (upper) and compound leaf petiole longitudinal sections (lower) between FZX and ZNX litchi cultivars.

FZX is a vigorous cultivar, with bigger leaf, compound leaf, and longer terminal bud. In contrast, ZNX is a dwarf cultivar, with small leaf, compound leaf, and shorter terminal bud (Table 1, Fig 1). According to the compound leaf petiole histological studies, huge differences were observed between FZX and ZNX. Compared with ZNX, the pith part and the ray cell is larger in FZX (Fig 1). Therefore, FZX and ZNX were selected to determine the transcriptional changes between vigorous and dwarf litchi cultivars in the present study.

**Table 1:** Comparasion of shoot and leaf length between ZNX and FZX.

Cultivar	Shoot length (cm)	Internode length (mm)	Leaf length (mm)	Leaf width (mm)
FZX	13.21 ± 2.17b	28.22 ± 4.61b	79.08 ± 7.93b	26.58 ± 3.53b
ZNX	25.60 ± 5.06a	48.72 ± 12.11a	163.45 ± 23.38a	49.09 ± 7.60a

Data were mean ± SE of three biological replicates. Different lowercase letters indicate significant difference at the 0.05 level based on Duncan multiple range test.

### RNA sequencing and transcript assembly

Twelve libraries were generated using the mRNA from four sample groups: FZX-leaves, FZX-apical-buds, ZNX-leaves and ZNX-apical-buds, each with three biological replicates. In addition, equal amounts of mRNA from the twelve samples were pooled, generating the library marked ‘pool’. These cDNA libraries were then subjected to Illumina deep sequencing. In total, 196,687,468 paired-end clean reads, each 150 bp in length were obtained from the pool (Table 2). Each library was represented by at least 13.5 million reads, a tag density sufficient for quantitative analysis of gene expression [44]. The reads were mapped to the reference sequence. There were at least 70% total mapped reads for each sample (Table 2).

**Table 2:** RNA-seq reads in twelve RNA-seq libraries.

Sample name	Number of input reads	Average input read length	Uniquely mapped reads number	Uniquely mapped reads%	Percent of reads mapped to multiple loci
FZX-apical-bud1	18,443,351	260	14241724	77.22	19.00
FZX-apical-bud2	23,002,507	255	17229221	74.90	18.85
FZX-apical-bud3	14,514,169	270	11070898	76.28	18.77
FZX-leaves1	20,029,366	273	15309985	76.44	19.65
FZX-leaves2	15,038,810	271	11355168	75.51	20.00
FZX-leaves3	16,136,701	271	12208298	75.66	19.62
ZNX-apical-bud1	12,918,190	270	9272820	71.78	21.60
ZNX-apical-bud2	16,079,578	269	11411651	70.97	22.15
ZNX-apical-bud3	15,923,038	269	11364189	71.37	21.95
ZNX-leaves1	15,635,653	270	11126550	71.16	21.90
ZNX-leaves2	13,732,058	269	9539948	69.47	18.71
ZNX-leaves3	15,234,047	271	10904961	71.58	21.87

All clean reads were *de novo* assembled into 150,867 transcripts using Trinity [36]. A total of 55,810 unigenes were obtained after redundancy removal using CD-hit [37]. The length of the unigenes ranged from 300–19,032 bp, with N50 of 2,376 bp. There were also 19,874 unigenes (35.61%), 17,584 unigenes (31.51%) in the length range of 1,000–2,000 bp and 18,352 unigenes (32.88%) with length >2000 bp (S1 Fig). Transdecoder from the Trinity package was used for CDS prediction. Approximately 48,660 (87.19%) out of all predicted CDS.

### Gene annotation and functional classification

**Fig 2:**
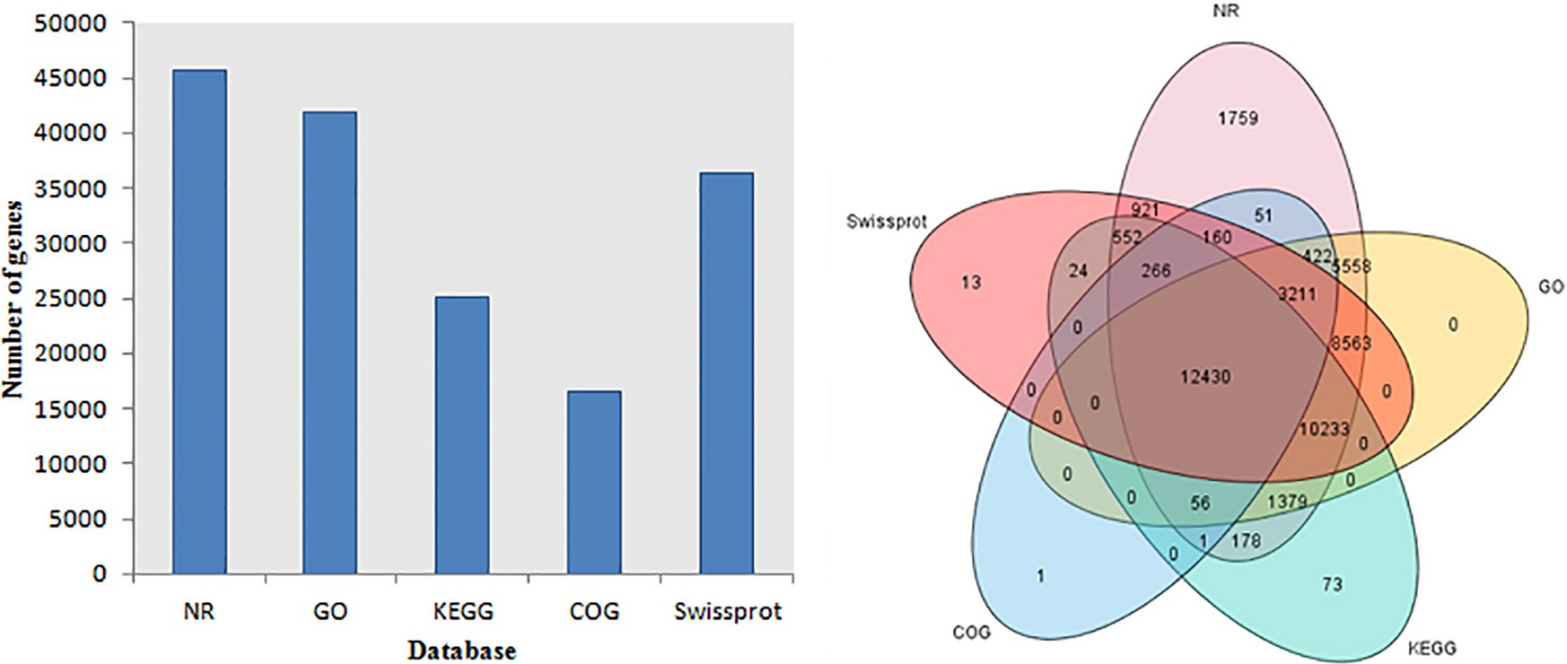
Distribution of annotation results of Unigenes in Nr, GO, KEGG, COG, Swissprot database.

**Fig 3:**
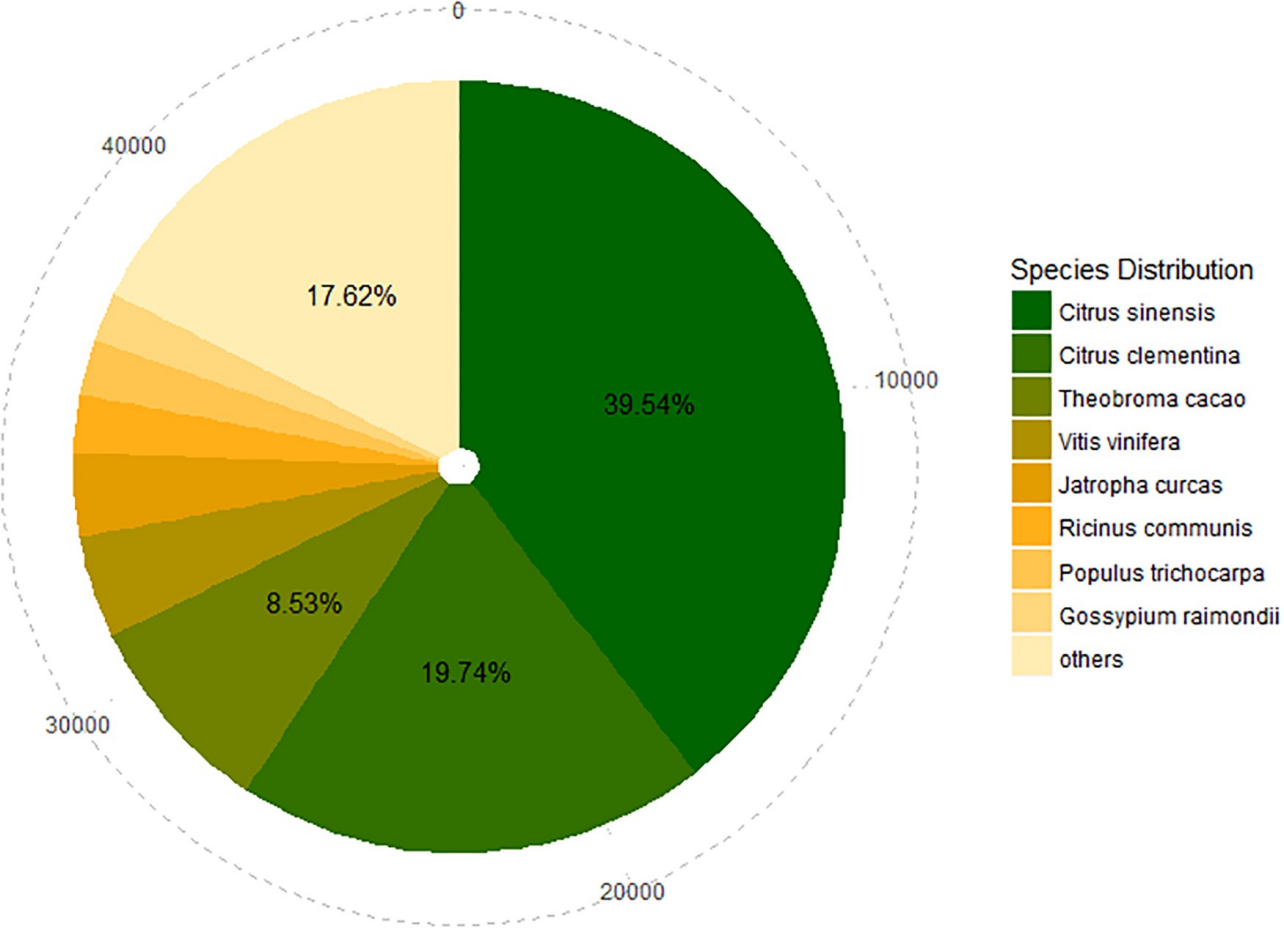
Species distribution of best blastx hits to NR database. Cumulative total numbers of unigenes annotated to NR were shown in the outermost circle with the dotted line. The size of the area was in proportion to the percentage of best blastx hits to the corresponding species.

All unigenes were annotated by query against various public databases (NR, COG, and KEGG). As a result, 45,740 (81.96% of 55,810) unigenes were matched to one or more of the databases (Fig 2). Of these unigenes, 18,084 (32.40%) were significantly similar to sequences of *Citrus sinensis*, and 9,026 (16.17%) and 3,899 (6.99%) unigenes showed high similarity to sequences of *Citrus clementina* and *Theobroma cacao*, respectively (Fig 3).

GO covers three domains: cellular component, molecular function and biological process. A total of 41,852 unigenes (74.99%) could be assigned to at least one GO term (S2 Fig), and detailed information of enriched GO terms was listed in S2 Table. Among these terms, the most representative terms in the biological process category were metabolic process, cellular process and single-organism process. ‘Cell’, ‘cell part’ and ‘organelle’ were the terms that dominated in the cellular component category. ‘Catalytic actibity’, ‘binding’ and ‘transporter activity’ were the most representative terms in the molecular function category. Only one unigene assigned in ‘behavior’.

All unigenes were aligned against the COG database for functional prediction and classification. In total, 16,599 (29.74% of 55,810) unigenes were assigned appropriate COG clusters, which could be classified into 25 functional categories (S3 Fig). Among them, the largest category was ‘General function prediction only’ (12.10% of 55,180), followed by ‘signal transduction mechanisms’ (9.71% of 55,180), ‘posttranslational modification, protein turnover, chaperones’ (4.29% of 55,180), ‘cell wall/membrane/envelope biogenesis’ (3.74% of 55,180), and ‘Transcription’ (3.61% of 55,180).

To further analyze the litchi transcripts, all the unigenes were analyzed with respect to the KEGG pathway database. In total, 25,192 unigenes were assigned to 139 KEGG pathways (S4 Fig). The pathways with the most representation among the unique sequences were the plant-pathogen interaction (4,504 unigenes), followed by plant hormone signal transduction (2,805 unigenes) and starch and sucrose metabolism (2,227 unigenes). Furthermore, some important pathways related to plant growth, development, morphogenesis and response to stress stimuli, including protein processing in endoplasmic reticulum (1,753 unigenes), carbon metabolism (1,677 unigenes), ribosome biogenesis in eukaryotes (1,639 unigenes), peroxisome(662 unigenes), ABC transporters (615 unigenes), zeatin biosynthesis (240 unigenes), citrate cycle (TCA cycle) (187 unigenes), photosynthesis (157 unigenes) and others, have also been annotated successfully.

### Differentially expressed genes (DEGs) between vigorous and dwarf litchi samples

**Fig 4:**
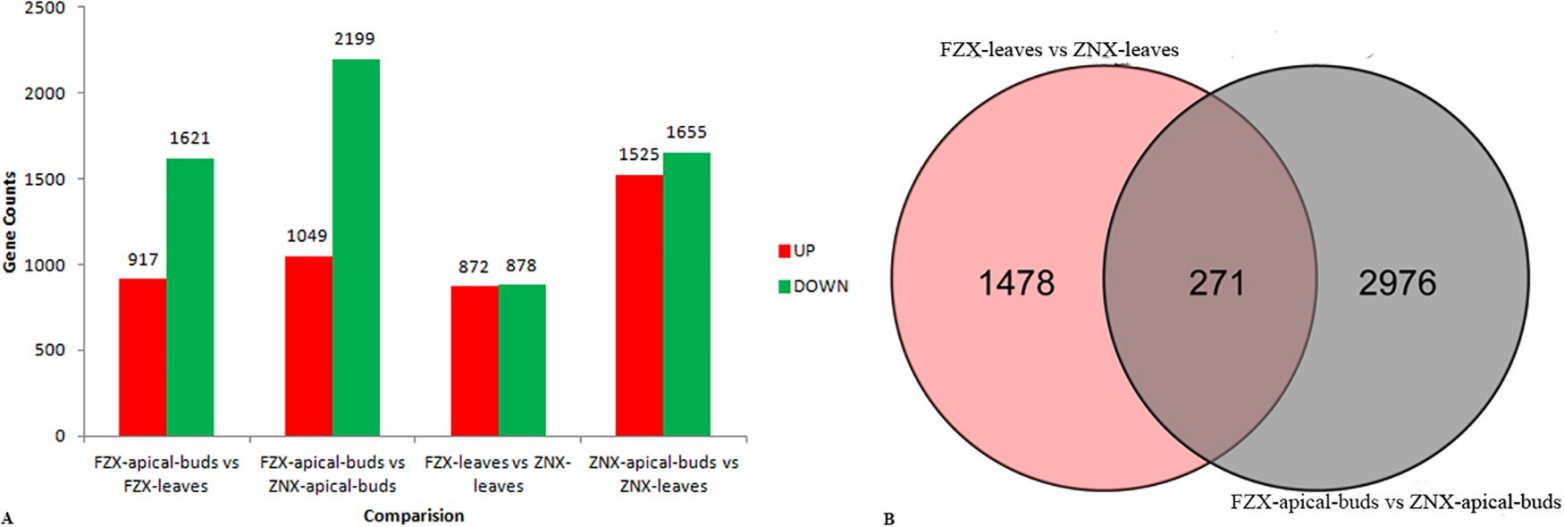
Differential gene expression profiles based on the library between vigorous and dwarf litchi samples. (A) The numbers of up- and down-regulated genes in comparisons of the FZX-apical-buds vs FZX-leaves, FZX-apical-buds vs ZNX-apical-buds, FZX-leaves vs ZNX-leaves, and ZNX-apical-buds vs ZNX-leaves litchi samples. (B) Venn diagram showing the comparison of differentially expressed genes between any two samples.

The expression patterns of unigenes between the vigorous and dwarf litchi samples were investigated. Pair wise comparison of the samples revealed many DEGs [|log2Ratio|≥1, false discovery rate (FDR)≤0.001] at the four libraries (FZX-leaves, FZX-apical-buds, ZNX-leaves, ZNX-apical-buds) (Fig 4). A total of 9,190 unigenes were found to be significantly differentially expressed in the pair-wise comparisons between any two samples. There were 2,538 DEGs (1,621 down-regulated and 917 up-regulated) between the FZX-leaves and FZX-apical-buds, and most of them were assigned to ‘plant hormone signal transduction’ KEGG pathways. There were 3,180 DEGs (1,655 down-regulated and 1,525 up-regulated) between ZNX-leaves and ZNX-apical-buds, and most of them were assigned to ‘biosynthesis of other secondary metabolites’ KEGG pathways. A total of 3,248 DEGs were detected between FZX-apical-buds and ZNX-apical-buds, with 2199 down-regulated and 1019 up-regulated. Regarding KEGG pathways, most of the DEGs were assigned to ‘genetic information process’ pathway. There were 1,750 DEGs (878 down-regulated and 872 up-regulated) between FZX-leaves and ZNX-leaves. Regarding KEGG pathways, most of the DEGs were assigned to ‘oxidative phosphorylation’ pathways.

Compared FZX-apical-buds vs ZNX-apical-buds with FZX-leaves vs ZNX-leaves, there were 1,478 DEGs only expressed in FZX-apical-buds vs ZNX-apical-buds, and 2,976 DEGs only expressed in FZX-leaves and ZNX-leaves. Moreover, 271 DEGs were both expressed in these two groups (S3 Table).

### DEGs related to phytohormone metabolism pathways

Phytohormone levels have been reported to be closely correlated with the dwarf and development of plants [20, 45]. To investigate the relationship between phytohormones and dwarf in litchi, the unigenes related to phytohormone metabolism were analyzed. In the present study, 65 unigenes in the abscisic acid (ABA) metabolism-related pathway, 115 unigenes in the brassinosteroid metabolism-related pathway, 39 unigenes in the ethylene metabolism-related pathway, 35 unigenes in the GA metabolism-related pathway and 97 unigenes in the cytokinins (CTK) metabolism-related pathway, were identified.

**Fig 5:**
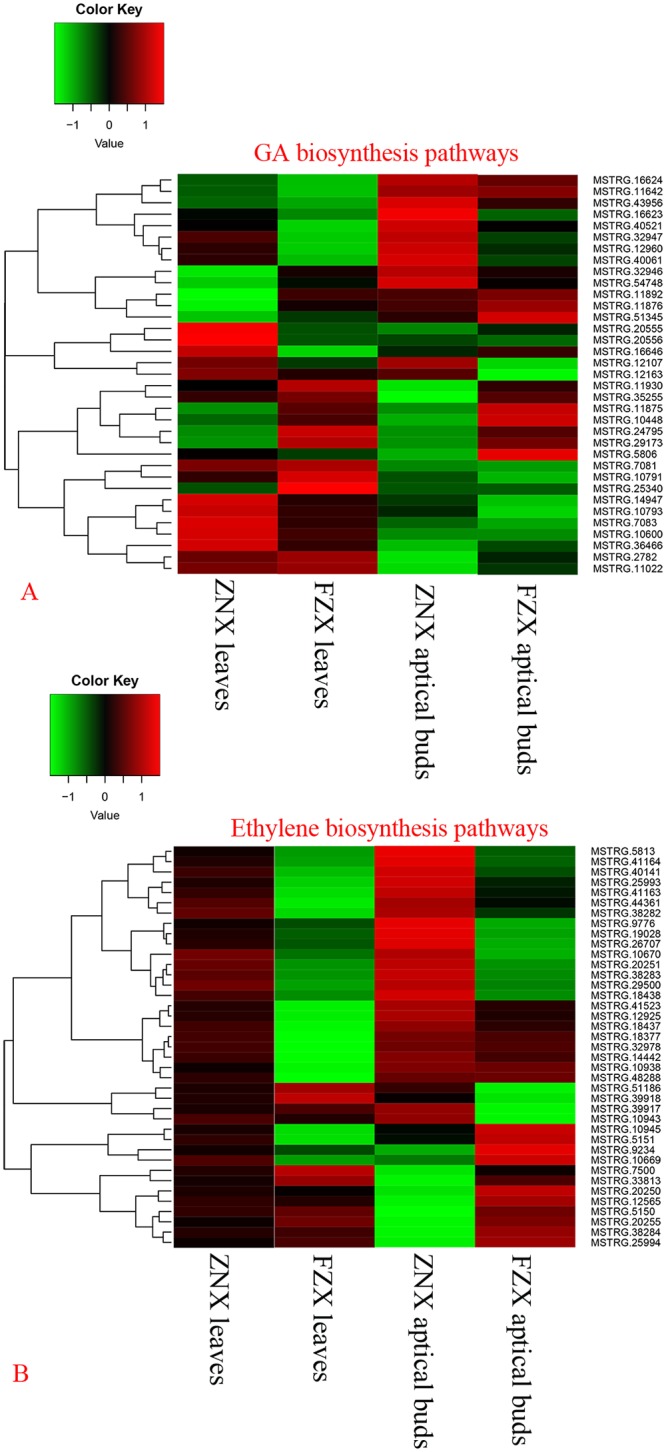
Heat map diagram of relative gene expression levels of DEGs related to GA (A) and ethylene (B) biosynthesis.

In this study, 35 unigenes involved in GA biosynthesis pathways were differentially expressed (Fig 5A). Among them, 18 unigenes were up-regulated in the ZNX-leaves, 20 unigenes were up-regulated in the FZX-leaves, 15 unigenes were up-regulated in the ZNX-aptical-buds, and 17 unigenes were up-regulated in the FZX-aptical-buds. In particular, *GA3ox* (MSTRG.5806, MSTRG.10448 and MSTRG.51345) were only up-regulated in FZX samples and *GA2ox* (MSTRG.12960, MSTRG.54748 and MSTRG.14947) were only up-regulated in ZNX samples (Fig 5A), indicating GA might play an important role in the huge difference between vigorous and dwarf litchi cultivars.

It is worth noting that the expression of ethylene related unigenes between FZX and ZNX samples had complete opposite trend, 39 unigenes involved in ethylene biosynthesis pathways were differentially expressed (Fig 5B). Almost all these DEGs were up-regulated in ZNX-leaves (16 SAMs, 12 ACO and 11 ACS) and 26 DEGs were up-regulated in ZNX-aptical-buds (9 SAMs, 8 ACO and 9 ACS). On the contrary, there were only 11 DEGs were up-regulated in FZX-leaves (7 SAMs, 3 ACO and 1ACS) and 19 DEGs were up-regulated FZX-aptical-buds (8 SAMs, 5 ACO and 6 ACS). Moreover, the expression of DEGs related to indole-3-acetic acid (IAA), CTK and BR metabolism had similar trend (data not shown).

### DEGs related to phytohormone signal transduction

**Fig 6:**
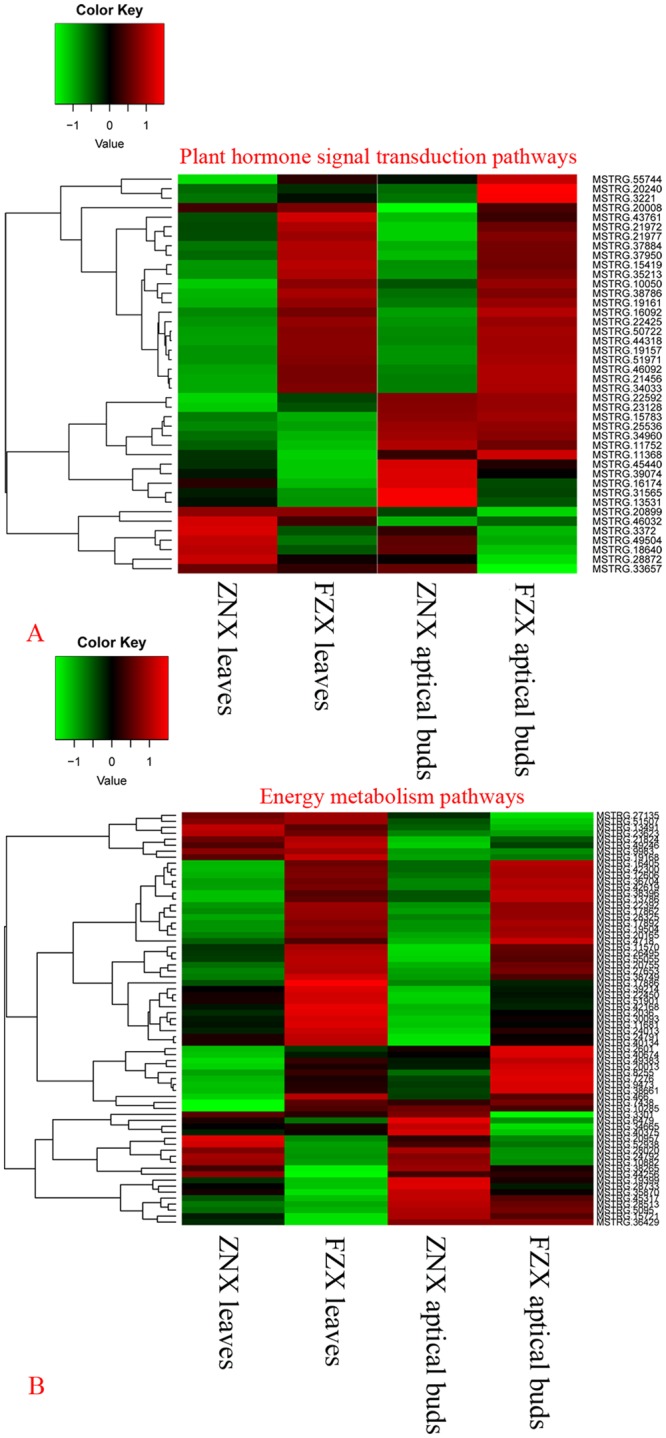
Heat map diagram of relative gene expression levels of DEGs related to plant hormone signal transduction pathways (A) and energy metabolism pathways (B).

A total of 42 DEGs were identified to involving in plant hormone signal transduction pathways (Fig 6A). Seven DEGs (MSTRG.16092, MSTRG.33657, MSTRG.37884, MSTRG.37950, MSTRG.44318, MSTRG.50722, MSTRG.55744) annotated as PROTEIN PHOSPHATASE 2C (PP2C) involved in ABA signal transduction were differentially expressed, and six out of the seven were up-regulated in FZX samples, and down-regulated in ZNX samples. One DEG (MSTRG.20008) annotated as serine/threonine-protein kinase SAPK was down-regulated in ZNX-aptical-buds. In the auxin-responsive pathway, six unigenes annotated as auxin response factors (ARFs) were differentially expressed. Among them, two unigenes (MSTRG.15783, MSTRG.34960) were only up-regulated in aptical-buds, two unigenes (MSTRG.46032, MSTRG.46092) were only up-regulated in FZX samples. The auxin receptor transport inhibitor response1 (TIR1) and auxin influx carrier protein gene was up-regulated in ZNX-aptical-buds. In addition, the two families of early auxin responsive genes, two out of three GH3 were up-regulated in aptical-buds and one SAUR was only up-regulated in FZX-aptical-buds. Of the DEGs related to CTK signaling, the levels of one type-A response regulator gene increased in ZNX-aptical-buds.

### DEGs related to energy metabolism pathways

In this study, a total of 69 DEGs were related to energy metabolism pathways (Fig 6B). Among them, there were 7 ATP synthase subunit unigenes, 5chlorophyll A/B binding protein unigenes, 2 fructose-1,6-bisphosphatase unigenes, 2 fructose-bisphosphate aldolase unigenes, 6 NADH dehydrogenase unigenes, 2 PSII core complex proteins unigenes, 2 PSI reaction center subunit II unigenes, and 2 plasma membrane ATPase-like unigenes. The great majority unigenes were up-regulated in FZX samples, especially in FZX leaves sample (Fig 6B).

### qRT-PCR validation of RNA-seq-based gene expression

qRT-PCR was carried out on 12 genes significantly differentially expressed as revealed by RNA-seq. They were MSTRG.15647, MSTRG.33946, MSTRG.46080, MSTRG.12329, MSTRG.26332, MSTRG.15171, MSTRG.26097, MSTRG.21401 and MSTRG.52145. Overall, the results of qRT-PCR were consistent with the RNA-seq data (Table 3). There was a high correlation between the qRT-PCR data and the RNA-seq data by linear regression analysis (S5 Fig).

**Table 3:** Confirmation of RNA-Seq expression profiles with qRT-PCR. *Lcactin* was used as reference gene to normalize gene expression levels under identical conditions.

Unigenes ID	Annotation	RNA-Seq (log2FoldChange)		qRCR (log2FoldChange)	
		FZX-apical-buds vs ZNX-apical-buds	FZX-leaves vs ZNX-leaves	FZX-apical-buds vs ZNX-apical-buds	FZX-leaves vs ZNX-leaves
MSTRG. 15647	alpha-expansin 3	-1.97	0.04	-1.79	1.99
MSTRG. 33946	phytochrome B	-3.02	-0.25	-0.81	-1.25
MSTRG. 46080	MYB-related protein 308-like	-2.74	0.22	-2.18	-3.06
MSTRG. 12329	Xylem bark cysteine peptidase	-2.46	0.51	-1.29	1.40
MSTRG. 15171	transcription factor AS1	3.89	0.06	2.67	0.18
MSTRG. 26332	cysteine proteinase	0.05	0.98	0.16	0.25
MSTRG. 26097	histone H1-like	-0.19	1.33	-0.69	-0.18
MSTRG. 21401	cysteine protease	-7.91	-0.75	-0.67	-1.32
MSTRG. 52145	DELLA protein GAIP-B-like	17.33	1.11	13.34	0.78
MSTRG.54748	gibberellin 2-oxidase	3.11	-3.00	2.33	-2.37
MSTRG.12960	gibberellin 2-oxidase	3.63	3.58	3.27	2.89
MSTRG.14947	gibberellin 2-oxidase	3.46	4.23	3.24	3.89

### Functional characterization of *LcGA2oxs* by stable expression in tobacco

**Fig 7:**
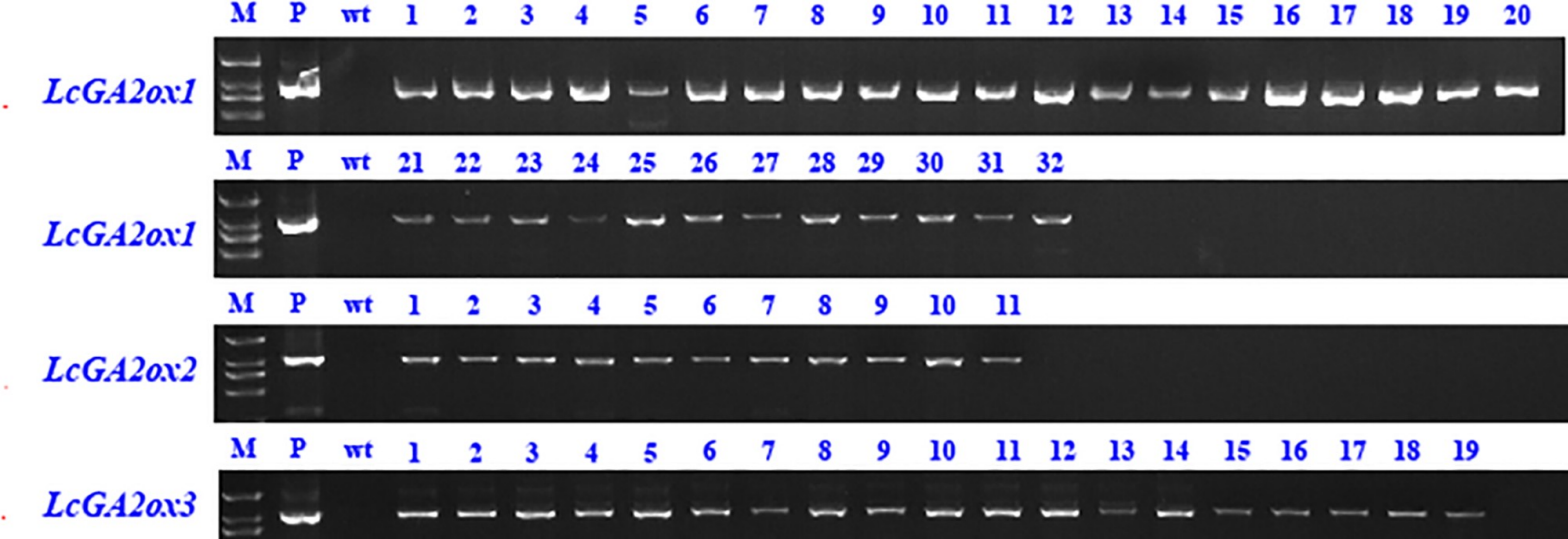
PCR analysis of *35S*::*LcGA2ox1* transgenic tobacco plants. M, Marker DL2000. P, positive control. wt, wild type.

**Fig 8:**
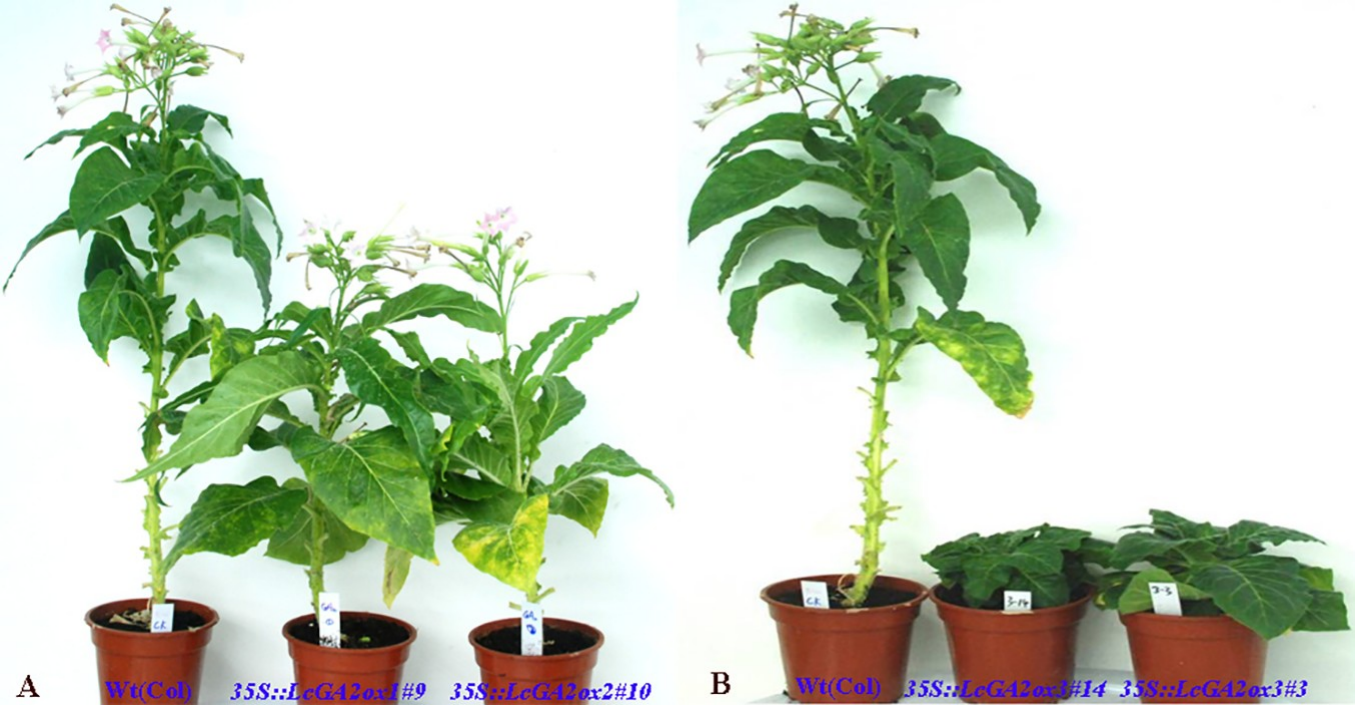
The phenotypes of transgenic tobacco lines ectopically expressing *LcGA2ox1*, *LcGA2ox2* and *LcGA2ox3*.

As mentioned above, *GA2ox* (MSTRG.12960, MSTRG.54748 and MSTRG.14947) were only up-regulated in ZNX samples (Fig 5A). To investigate the role of *LcGA2ox* in litchi dwarfism, their function was investigated by stable expression in tobacco. Three *LcGA2ox* genes, *LcGA2ox1* (MSTRG.54748), *LcGA2ox2* (MSTRG.12960), and *LcGA2ox3* (MSTRG.14947), under the expression of the 35S promoter were transformed into tobacco. The existence of introduced *LcGA2ox* genes was confirmed by PCR. Thirty-two, eleven, and nineteen independent PCR-positive transgenic T1 plants were obtained for *LcGA2ox1*, *LcGA2ox2*, and *LcGA2ox3*, respectively (Fig 7). All transgenic plants were phenotypically distinguishable from wild type plants (Fig 8). Firstly, these *35S*::*LcGA2ox* transgenic plants were smaller than wild type plants (Fig 8A). Secondly, *35S*::*LcGA2ox* transgenic plants flowered later than the wild type plants. When the wild type plants flowered, they were over 80 cm high, whereas *35S*::*LcGA2ox* transgenic plants were only 6 cm high (Fig 8B). As many lines had a typical dwarf phenotype, three independent transgenic lines of each *35S*::*LcGA2ox* transgenic plants with a significant change of plant height were selected for further investigation after 6 months of planting. As shown in Table 4, six phenotypes including days to flowering, plant height, stem diameter, leaf area, internode length, and internode number were measured. In particular, the plant height of transgenic tobacco *35S*::*LcGA2ox3#3* was only 3.28 cm. Besides plant height, leaf area was smaller in in transgenic plants compared with the wildtype (Table 4). These results showed that ectopic overexpression of *LcGA2ox* genes in tobacco leads to the dwarf phenotype in transgenic plants.

**Table 4:** Characteristics of transgenic lines ectopically expressing *LcGA2ox1*, *LcGA2ox2* and *LcGA2ox3*.

Line	No. of plants	Days to flowering	Plant height (cm)	Stem diameter (mm)	Leaf area(cm^2^)	Internode length(cm)	Internode number
W. T.	5	79.72 ± 15.34 b	32.41 ± 3.76 a	9.02 ± 0.27 b	207.90 ± 14.65 a	1.90 ± 0.50 a	15.60 ± 1.15 bc
*35S*::*LcGA2ox1*							
#9	5	106.72 ± 19.91 ab	20.88 ± 1.43b	7.65 ± 0.76 c	132.47 ± 36.26 bc	1.30 ± 0.35 bc	17.60 ± 1.53 b
#10	4	104.36 ± 11.99 ab	21.46 ± 3.01b	6.92 ± 0.80 cd	121.09 ± 0.45 bc	1.43 ± 0.21 b	15.25 ± 2.52 bc
#11	5	103.33 ± 15.69 ab	21.72 ± 0.84 b	6.18 ± 0.20 d	103.43 ± 4.64 bc	1.55 ± 0.13 ab	13.30 ± 1.15 c
*35S*::*LcGA2ox2*							
#7	5	109.27 ± 12.93 a	20.96 ± 2.78 b	7.80 ± 0.57 c	141.30 ± 45.89 bc	1.30 ± 0.21 bc	17.60 ± 1.53 b
#10	5	112.38 ± 12.97 a	18.75 ± 1.96 bc	8.78 ± 0.72 b	136.07 ± 25.03 bc	0.85 ± 0.13 cd	21.30 ± 2.08 a
#11	5	112.17 ± 18.54 a	16.36 ± 2.60 c	9.92 ± 0.31 a	152.42 ± 48.84 bc	1.27 ± 0.39 bc	13.30 ± 2.52 c
*35S*::*LcGA2ox3*							
#3	5	120.76 ± 14.11 a	3.28 ± 1.30 d	3.93 ± 0.29 e	94.63 ± 38.98 c	0.25 ± 0.04 e	15.30 ± 2.52 bc
#14	4	114.59 ± 19.23 a	6.22 ± 0.85 d	7.30 ± 0.43 c	160.99 ± 1.51 ab	0.34 ± 0.06 e	17.50 ± 1.53 b
#18	4	117.69 ± 12.42 a	6.54 ± 1.65 d	7.18 ± 0.21 c	119.04 ± 31.01 bc	0.43 ± 0.04 de	15.25 ± 0.58 bc

Data were mean ± SE of three biological replicates. Different lowercase letters indicate significant difference at the 0.05 level among 8 sampling sites based on Duncan multiple range test.

## Discussion

Dwarfism is a desirable characteristic for many agricultural plants. Dwarf can reduce lodging and increase harvest index in grain crops. The major factor of the success of the Green Revolution was the dwarf wheat (*Triticum aestivum*) and rice (*Oryza sativa*) cultivars breeding [46]. In the modern fruit culture and production, dwarfing and close planting are very important targets. Farmers often use dwarfing rootstocks or dwarf cultivars to develop suitable germplasms and achieve more profits [47]. Many dwarfing apple rootstocks are widely used in production, but these resources are limited in litchi cultivation [48].

In our previous study, ZNX is identified as a dwarf litchi germplasm [29]. In the present study, the anatomical observation supported this view (Fig 1). Therefore, ZNX might be an excellent germplasm to study the mechanisms of dwarf in litchi. In this study, transcriptome analysis was adopted to understand the global molecular events of differentially expressed genes between vigorous (FZX) and dwarf (ZNX) litchi cultivars. RNA-Seq technology was used to profile the litchi buds and leaves of vigorous and dwarf litchi cultivars transcriptome on the Illumina HiSeqTM 2500 platform, and approximately 197 million paired-end clean reads were obtained. 45,740 out of 55,810 unigenes were successfully annotated against public databases, suggesting their relatively conserved functions. A total of 9,190 unigenes were found to be significantly differentially expressed in the pair-wise comparisons between any two samples (Fig 4), which would provide useful information on the litchi dwarf mechanism.

Several hypotheses have been suggested to explain dwarf mechanisms in plant, such as anatomical [49–51], nutritional [52–54], and hormonal [55–58].

### Anatomical characteristics

Several indicators, such as palisade/spongy ratio, wood/bark ratio, vessel diameter and vessel density, have been used to distinguish dwarfing rootstocks [31, 49, 50, 59, 60]. In this study, we also found there were huge differences between vigorous and dwarf litchi cultivars (Table 1, Fig 1). Compared with FZX, the pith part and the ray cell of ZNX is smaller, indicating that ZNX is a potential dwarf litchi cultivar.

### Phytohormone related pathways

Among the different factors responsible for plant dwarfism, phytohormone was the main reason. GA and BR have been extensively studied in determining plant height [20, 45]. In plants, the flux of bioactive GAs is regulated by the balance between their biosynthesis rates and deactivation. 2-oxidation pathway has been suggested to be a major mechanism for GA inactivation [61–63]. Thus, overexpression of *GA2ox* results in dwarfism [64–66]. On the other hand, the suppression of *GA2ox* expression leads to tall and slender phenotypes [63, 67]. In this study, 35 unigenes involved in GA biosynthesis pathways were differentially expressed (Fig 5A). In particular, *GA3ox* (MSTRG.5806, MSTRG.10448 and MSTRG.51345) were only up-regulated in FZX samples *GA2ox* (MSTRG.12960, MSTRG.14947 and MSTRG.10600) were only up-regulated in ZNX samples. Additional, ectopic overexpression of *LcGA2ox* genes in tobacco resulted in a dominant dwarf and later flower phenotype (Fig 8). Our results indicate that the differentially expressed genes involved in GA biosynthesis pathways may result in the difference in growth vigor between FZX and ZNX.

There are few reports about ethylene involving in dwarfism. However, the expression of ethylene related unigenes between FZX and ZNX samples had complete opposite trend in the present study (Fig 5B). Amost all unigenes were up-regulated in ZNX-leaves, including 16 SAMs, 12 ACO and 11 ACS. The correlation between ethylene and litchi dwarfing is worth further research. CTK and auxins play particularly significant role in regulating plant growth and development [67–70]. In this study, most CTK and IAA related unigenes were up-regulated in the aptical-buds samples. Besides, the up-regulated unigenes in FZX were more than that in ZNX.

### Energy metabolism pathways

During the process of photosynthesis, plants convert light energy from the sun into chemical energy stored in molecules. The vigorous plants need stronger energy metabolism to support plant growth and development. Increasing studies suggested that sugars not just as nutrients, but also as signal molecules sensing nutrient status and coordinating plant growth and development accordingly [71–75]. In this study, 69 DEGs related to energy metabolism pathways were found (Fig 6B). Moreover, the great majority DEGs were up-regulated in FZX (the vigorous cultivar) samples, especially in FZX leaves sample, indicating the vigorous FZX need stronger energy metabolism to support vigorous growth.

## Conclusions

Transcriptome analysis of differentially expressed genes between FZX (vigorous cultivar) and ZNX (dwarf cultivar) revealed interesting genes that might provide some clues revealing the mechanisms of litchi dwarfism. The expression of genes involved in phytohormone related pathways and energy metabolism pathways exist huge differences between vigorous and dwarf litchi cultivars, especially the GA and ethylene related genes. Ectopic overexpression of *LcGA2ox* genes leads to the dwarf phenotype in transgenic tobacco. In addition, GA and ethylene might interact with other hormone signaling pathways, such as auxin, ABA, and CTK, forming a complex network to regulate plant growth and development.

## Supporting information

S1 Table Primer sequence of genes. (DOC)

S2 Table The list of enriched GO terms. (XLS)

S3 Table The list of DEGs both expressed in FZX-apical-buds vs ZNX-apical-buds and FZX-leaves vs ZNX-leaves. (XLS)

S1 Fig Length distribution of unigenes. The x-axis denoted the length range of all groups. The y-axis denoted the number of unigenes in each group.
(TIF)

S2 Fig Gene ontology classification of unigenes. The number of gene GO terms in each functional subcategory was presented as the percentage of GO terms for that subcategory out of the total GO terms.
(TIF)

S3 Fig COG classification of unigenes. The y-axis denoted the number of unigenes in each group. The x-axis denoted the functional description of each group. Details were shown in the right part of the graph.
(TIF)

S4 Fig KEGG classification of unigenes. The y-axis denoted the number of unigenes in each group. The x-axis denoted subclass of KEGG.
(TIF)

S5 Fig Correlation between qRT-PCR and data obtained from transcriptome analysis. The real-time PCR log2 values (x-axis) were plotted against colorationstages (y-axis). **indicates a significant difference at p ≤ 0.01.
(TIF)

## References

[1] Hollender CA, Hadiarto T, Srinivasan C, Scorza R, Dardick C. A brachytic dwarfism trait (*dw*) in peach trees is caused by a nonsense mutation within the gibberellic acid receptor *PpeGID1c*. New Phytol. 2016; 210: 227–239. 10.1111/nph.13772 26639453 10.1111/nph.13772

[2] Byrne DH. Springer In: Badenes ML, Byrne DH, editors. Fruit Breeding Trends in fruit breeding; 2012 pp. 3–36.

[3] Webster T. Dwarfing rootstocks: past, present and future. Compact Fruit Tree 2002; 35: 67–72.

[4] Miller SR, Heeney HB, Nelson SH. Studies on apple rootstock selections relating respiration rates to an anatomical predicting dwarfness. Can. J. Plant Sci. 1961; 41: 221–226.

[5] Larsen FE, Fritts R Jr. Rootstock influence (1965–1980) on yield, yield efficiency and tree size of ‘Bartlett’ and ‘d’Anjou’ pear. Sci. Hortic. 1984; 24: 271–278.

[6] Webster AD. Rootstock and interstock effects on deciduous fruit tree vigour, precocity, and yield productivity. New Zeal. J. Crop Hort. 1995; 23: 373–382.

[7] Facteau TJ, Chestnut NE, Rowe KE. Tree, fruit size and yield of ‘Bing’ sweet cherry as influenced by rootstock, replant area, and training system. Sci. Hortic. 1996; 67: 13–26.

[8] Webster AD. Vigour mechanisms in dwarfing rootstocks for temperate fruit trees. Acta Hort. 2004; 658: 29–41.

[9] Olesen T, Menzel CM, McConchie CA, Wiltshire N. Pruning to control tree size, flowering and production of litchi. Sci. Hortic. 2013; 156: 93–98.

[10] Davis TD, Curry EA, Steffens GL. Chemical regulation of vegetative growth. Crit. Rev. Plant Sci. 1991; 10: 151–188.

[11] Medjdoub R, Val J, Blanco A. Prohexadione-Ca inhibits vegetative growth of ‘Smoothee Golden Delicious’ apple trees. Sci. Hortic. 2004; 101: 243–253.

[12] Hollender CA, Dardick C. Molecular basis of angiosperm tree architecture. New Phytol. 2015; 206: 541–556. 10.1111/nph.13204 25483362 10.1111/nph.13204

[13] Kenis K, Keulemans J. Study of tree architecture of apple (*Malus*× *domestica* Borkh.) by QTL analysis of growth traits. Mol. Breeding 2007; 19: 193–208.

[14] Segura V, Denancé C, Durel CE, Costes E. Wide range QTL analysis for complex architectural traits in a 1-year-old apple progeny. Genome 2007; 50: 159–171. 10.1139/g07-002 17546081 10.1139/g07-002

[15] Habu T, Yamane H, Naito I, Nishiyama S, Nonaka A, Kawai T, Yamada H, Tao R. Differences in physiological characteristics and gene expression levels in fruits between Japanese persimmon (*Diospyros kaki* Thunb.) ‘Hiratanenashi’ and its small fruit mutant ‘Totsutanenashi’. Horticult. J. 2016; 85: 306–314.

[16] Chen J, Xie J, Duan Y, Hu H, Li W. Genome-wide identification and expression profiling reveal tissue-specific expression and differentially-regulated genes involved in gibberellin metabolism between Williams banana and its dwarf mutant. BMC Plant Biol. 2016; 16: 123 10.1186/s12870-016-0809-1 27234596 10.1186/s12870-016-0809-1 PMC4884393

[17] Peng J, Richards DE, Hartley NM, Murphy GP, Devos KM, Flintham JE, Beales J, Fish LJ, Worland AJ, Pelica F, Sudhakar D, Christou P, Snape JW, Gale MD, Harberd NP. ‘Green revolution’ genes encode mutant gibberellin response modulators. Nature 1999; 400: 256–261. 10.1038/22307 10421366 10.1038/22307

[18] Topp S.H., Rasmussen S.K. Evaluating the potential of *SHI* expression as a compacting tool for ornamental plants. *Plant Sci*. 2012, 187, 19–30. 10.1016/j.plantsci.2012.01.007 22404829 10.1016/j.plantsci.2012.01.007

[19] Wang Y, Zhao J, Lu W, Deng D. Gibberellin in plant height control: old player, new story. Plant Cell Rep. 2017; 36: 391–398. 10.1007/s00299-017-2104-5 28160061 10.1007/s00299-017-2104-5

[20] Yamaguchi S. Gibberellin metabolism and its regulation. Annu. Rev. Plant Biol. 2008; 59: 225–251. 10.1146/annurev.arplant.59.032607.092804 18173378 10.1146/annurev.arplant.59.032607.092804

[21] Hedden P, Phillips AL. Gibberellin metabolism: new insights revealed by the genes. Trends Plant Sci. 2000; 5: 523–530. 11120474 10.1016/s1360-1385(00)01790-8

[22] Bulley SM, Wilson FM, Hedden P, Phillips AL, Croker SJ, James DJ. Modification of gibberellin biosynthesis in the grafted apple scion allows control of tree height independent of the rootstock. Plant Biotech. J. 2005; 3: 215–223. 10.1111/j.1467-7652.2005.00119.x 17173621

[23] Schomburg FM, Bizzell CM, Lee DJ, Zeevaart JA, Amasino RM. Overexpression of a novel class of gibberellin 2-oxidases decreases gibberellin levels and creates dwarf plants. Plant Cell 2003; 15: 151–163. 10.1105/tpc.005975 12509528 10.1105/tpc.005975 PMC143488

[24] Zhu LH, Li XY, Welander M. Overexpression of the *Arabidopsis gai* gene in apple significantly reduces plant size. Plant Cell Rep. 2008; 27: 289–296. 10.1007/s00299-007-0462-0 17932677 10.1007/s00299-007-0462-0

[25] Li JG. The Litchi. Beijing: China Agriculture Press; 2008.

[26] Zhang, Z.W. Guangdong Academy of Agricultural Science. In: Zhang ZW, Yuan PY, Wang BQ, Qiu YP, Li JS, editors. Litchi: Pictorial Narration of Cultivation. China, the native home of litchi; 1997. pp. 12–17.

[27] Menzel C. The physiology of growth and cropping in lychee. In Proceedings of 1st International Symposium on litchi and longan, Guangzhou, China, 2000. pp. 37.

[28] Waite GK, Menzel CM. Litchi and Logan: botany, production and uses. CABI publishing; 2005.

[29] Zhang Y, Lu B, Pan L, Wang Y, Hu G, Hu Y, Wang H, Liu C. Dwarfing related mechanisms of dwarf cultivars in *Litchi chinensis*. J. Fruit Sci. 2011; 28: 624–629.

[30] Tombesi S, Johnson RS, Day KR, DeJong TM. Relationships between xylem vessel characteristics, calculated axial hydraulic conductance and size-controlling capacity of peach rootstocks. Ann. Bot. 2010; 105: 327–331. 10.1093/aob/mcp281 19939979 10.1093/aob/mcp281 PMC2814754

[31] Zorić L, Ljubojević M, Merkulov L, Luković J, Ognjanov V. Anatomical characteristics of cherry rootstocks as possible preselecting tools for prediction of treevigor. J. Plant Growth Regul. 2012; 31: 320–331.

[32] Chen Z, Zhao J, Hu F, Qin Y, Wang X, Hu G. Transcriptome changes between compatible and incompatible graft combination of *Litchi chinensis* by digital gene expression profile. Sci. Rep. 2017; 7: 3954 10.1038/s41598-017-04328-x 28638079 10.1038/s41598-017-04328-x PMC5479835

[33] Bolger AM, Lohse M, Usadel B. Trimmomatic: a flexible trimmer for Illumina sequence data. Bioinformatics 2014; 30: 2114–2120. 10.1093/bioinformatics/btu170 24695404 10.1093/bioinformatics/btu170 PMC4103590

[34] Quast C, Pruesse E, Yilmaz P, Gerken J, Schweer T, Yarza P, Peplies J, Glöckner FO. The SILVA ribosomal RNA gene database project: improved data processing and web-based tools. Nucl. Acids Res. 2013; 41 (D1): D590–D596. 23193283 10.1093/nar/gks1219 PMC3531112

[35] Li H, Durbin R. Fast and accurate short read alignment with Burrows-Wheeler transform. Bioinformatics 2009; 25: 1754–1760. 10.1093/bioinformatics/btp324 19451168 10.1093/bioinformatics/btp324 PMC2705234

[36] Grabherr MG, Haas BJ, Yassour M, Levin JZ, Thompson DA, Amit I, Adiconis X, Fan L, Raychowdhury R, Zeng Q, Chen Z, Mauceli E, Hacohen N, Gnirke A, Rhind N, di Palma F, Birren BW, Nusbaum C, Lindblad-Toh K, Friedman N, Regev A. Full-length transcriptome assembly from RNA-Seq data without a reference genome. Nat. Biotechnol. 2011; 29: 644–652. 10.1038/nbt.1883 21572440 10.1038/nbt.1883 PMC3571712

[37] Fu L, Niu B, Zhu Z, Wu S, Li W. CD-HIT: accelerated for clustering the next-generation sequencing data. Bioinformatics 2012; 28: 3150–3152. 10.1093/bioinformatics/bts565 23060610 10.1093/bioinformatics/bts565 PMC3516142

[38] Trapnell C, Williams BA, Pertea G, Mortazavi A, Kwan G, van Baren MJ, Salzberg SL, Wold BJ, Pachter L. Transcript assembly and quantification by RNA-Seq reveals unannotated transcripts and isoform switching during cell differentiation. Nat. Biotechnol. 2010; 28: 511–515. 10.1038/nbt.1621 20436464 10.1038/nbt.1621 PMC3146043

[39] Anders S, Huber W. Differential expression analysis for sequence count data. Genome Biol. 2010; 11: R106 10.1186/gb-2010-11-10-r106 20979621 10.1186/gb-2010-11-10-r106 PMC3218662

[40] Haynes W. Springer In: Dubitzky W, Wolkenhauer O, Cho KH, Yokota H, eds. Encyclopedia of Systems Biology Benjamini–Hochberg Method; 2013.

[41] Supek F, Bošnjak M, Škunca N, Šmuc T. REVIGO summarizes and visualizes long lists of gene ontology terms. PLoS One 2011; 6: e21800 10.1371/journal.pone.0021800 21789182 10.1371/journal.pone.0021800 PMC3138752

[42] Livak KJ, Schmittgen TD. Analysis of relative gene expression data using real-time quantitative PCR and the 2(-Delta DeltaC(T)) Method. Methods 2001; 25: 402–408. 10.1006/meth.2001.1262 11846609 10.1006/meth.2001.1262

[43] Chen G, Hackett R, Walker D, Taylor A, Lin Z, Grierson D. Identification of a specific isoform of tomato lipoxygenase (TomloxC) involved in the generation of fatty acid-derived flavor compounds. Plant Physiol. 2004; 136: 2641–2651. 10.1104/pp.104.041608 15347800 10.1104/pp.104.041608 PMC523329

[44] Morin RD, O’Connor MD, Griffith M, Kuchenbauer F, Delaney A, Prabhu AL, Zhao Y, McDonald H, Zeng T, Hirst M, Eaves CJ, Marra MA. Application of massively parallel sequencing to microRNA profiling and discovery in human embryonic stem cells. Genome Res. 2008; 18: 610–621. 10.1101/gr.7179508 18285502 10.1101/gr.7179508 PMC2279248

[45] Fujioka S, Yokota T. Biosynthesis and metabolism of brassinosteroids. Annu. Rev. Plant Biol. 2003; 54: 137–164. 10.1146/annurev.arplant.54.031902.134921 14502988 10.1146/annurev.arplant.54.031902.134921

[46] Khush GS. Green revolution: the way forward. Nat. Rev. Genet. 2001; 2: 815–822. 10.1038/35093585 11584298 10.1038/35093585

[47] Fideghelli C, Sartori A, Grassi F. Fruit tree size and architecture. Acta Hortic. 2003; 662: 279–293.

[48] Elkins R, Bell R, Einhorn T. Needs assessment for future US pear rootstock research directions based on the current state of pear production and rootstock research. J. Am. Pomol. Soc. 2012; 66: 153–163.

[49] Olmstead MA, Lang NS, Ewers FW, Owens SA. Xylem vessel anatomy of sweet cherries grafted onto dwarfing and nondwarfing rootstocks. J. Am. Soc. Hortic. Sci. 2006; 131: 577–585.

[50] Trifilà P, Gullo MAL, Nardini A, Pernice F, Salleo S. Rootstock effects on xylem conduit dimensions and vulnerability to cavitation of *Olea europaea* L. Trees 2007; 21: 549–556.

[51] Tombesi S, Almehdi A, DeJong TM. Phenotyping vigour control capacityof new peach rootstocks by xylem vessel analysis. Sci. Hortic. 2011; 127: 353–357.

[52] Jones OP. Effect of dwarfing interstocks on xylem sap composition in apple trees: effect on nitrogen, potassium, phosphorus, calcium and magnesium content. Ann. Bot. 1976; 40: 1231–1235.

[53] Schechter I, Elfving DC, Proctor JTA. Apple tree canopy development and photosynthesis as affected by rootstock. Can. J. Bot. 1991; 69: 295–300.

[54] Fallahi E, Chun IJ, Neilsen GH, Colt WM. Effects of three rootstocks on photosynthesis, leaf mineral nutrition, and vegetative growth of BC-2 Fuji apple trees. J. Plant Nutr. 2001; 24: 827–834.

[55] Richards D, Thompson WK, Pharis RP. The influence of dwarfing interstocks on the distribution and metabolism of xylem-applied [3H] gibberellin A4 in apple. Plant Physiol. 1986; 82: 1090–1095. 16665139 10.1104/pp.82.4.1090 PMC1056263

[56] Kamboj JS, Blake PS, Quinlan JD, Baker DA. Identification and quantitation by GC–MS of zeatin and zeatin riboside in xylem sap from rootstock and scion of grafted apple trees. Plant Growth Regul. 1999; 28: 199–205.

[57] Kamboj JS, Browning G, Blake PS, Quinlan JD, Baker DA. GC–MS-SIM analysis of abscisic acid and indole-3-acetic acid in shoot bark of apple rootstocks. Plant Growth Regul. 1999; 28: 21–27.

[58] Sorce C, Massai R, Picciarelli P, Lorenzi R. Hormonal relationships in xylem sap of grafted and ungrafted *Prunus* rootstocks. Sci. Hortic. 2002; 93: 333–342.

[59] Kurian RM, Iyer CPA. Stem anatomical characters in relation to tree vigourin mango (*Mangifera indica* L.). Sci. Hortic. 1992; 50: 245–253.

[60] Hajagos A, Végvári G. Investigation of tissue structure and xylem anatomyof eight rootstocks of sweet cherry (*Prunus avium* L.). Trees 2013; 27: 53–60.

[61] Thomas SG, Phillips AL, Hedden P. Molecular cloning and functional expression of gibberellin 2-oxidases, multifunctional enzymes involved in gibberellin deactivation. PNAS 1999; 96: 4698–4703. 10200325 10.1073/pnas.96.8.4698 PMC16395

[62] MacMillan J. Occurrence of gibberellins in vascular plants,fungi, and bacteria. J. Plant Growth Regul. 2001; 20: 387–442. 10.1007/s003440010038 11986764 10.1007/s003440010038

[63] Rieu I, Eriksson S, Powers SJ, Gong F, Griffiths J, Woolley L, Benlloch R, Nilsson O, Thomas SG, Hedden P, Phillips AL. Genetic analysis reveals that C19-GA 2-oxidation is a major gibberellin inactivation pathway in Arabidopsis. Plant Cell 2008; 20: 2420–2436. 10.1105/tpc.108.058818 18805991 10.1105/tpc.108.058818 PMC2570722

[64] Sakamoto T, Morinaka Y, Ishiyama K, Kobayashi M, Itoh H, Kayano T, Iwahori S, Matsuoka M, Tanaka H. Genetic manipulation of gibberellin metabolism in transgenic rice. Nat. Biotechnol. 2003; 21: 909–913. 10.1038/nbt847 12858182 10.1038/nbt847

[65] Lee DJ, Zeevaart JAD. Molecular cloning of *GA 2-Oxidase3* from Spinach and its ectopic expression in *Nicotiana sylvestris*. Plant Physiol. 2005; 138: 243–254. 10.1104/pp.104.056499 15821147 10.1104/pp.104.056499 PMC1104179

[66] Hu YX, Tao YB, Xu ZF. Overexpression of *Jatropha Gibberellin 2-oxidase 6* (*JcGA2ox6*) induces dwarfism and smaller leaves, flowers and fruits in *Arabidopsis* and *Jatropha*. *Front*. Plant Sci. 2017; 8: 2103 10.3389/fpls.2017.02103 29312375 10.3389/fpls.2017.02103 PMC5733080

[67] Martin DN, Proebsting WM, Hedden P. The *SLENDER* gene of pea encodes a gibberellin 2-oxidase. Plant Physiol. 1999; 121: 775–781. 10557225 10.1104/pp.121.3.775 PMC59439

[68] Soumelidou K, Morris DA, Battey NH, Barnett JR, John P. Auxin transport capacity in relation to the dwarfing effect of apple rootstocks. J. Hortic. Sci. 1994; 69: 719–725.

[69] Michalczuk L. Indole-3-acetic acid level in wood, bark and cambial sap of apple rootstocks differing in growth vigour. Acta Physiol. Plant 2002; 24: 131–136.

[70] Peng J, Peng FT, Wei SC. Effect of nitrogen forms on *IPT3* expression and hormone content of Pingyitiancha (*Malus hupenensis* Rehd.). Sci. Agric. Sinica 2008; 41: 3716–3721.

[71] Rolland F, Baena-Gonzalez E, Sheen J. Sugar sensing and signaling in plants: conserved and novel mechanisms. Annu. Rev. Plant Biol. 2006; 57: 675–709. 10.1146/annurev.arplant.57.032905.105441 16669778 10.1146/annurev.arplant.57.032905.105441

[72] Eveland AL, Jackson DP. Sugars, signalling, and plant development. J. Exp. Bot. 2012; 63: 3367–3377. 10.1093/jxb/err379 22140246 10.1093/jxb/err379

[73] Moghaddam MRB, Ende WVd. Sugars, the clock and transition to flowering. Front. Plant Sci. 2013; 4: 22 10.3389/fpls.2013.00022 23420760 10.3389/fpls.2013.00022 PMC3572515

[74] Dobrenel T, Marchive C, Azzopardi M, Clément G, Moreau M, Sormani R, Robaglia C, Meyer C. Sugar metabolism and the plant target of rapamycin kinase: a sweet operaTOR? Front. Plant Sci. 2013; 4: 93 10.3389/fpls.2013.00093 23641244 10.3389/fpls.2013.00093 PMC3640205

[75] Lastdrager J, Hanson J, Smeekens S. Sugar signals and the control of plant growth and development. J. Exp. Bot. 2014; 65: 799–807. 10.1093/jxb/ert474 24453229 10.1093/jxb/ert474

