# Supplementary material for: Differential gene expression between the vigorous and dwarf litchi cultivars based on RNA-Seq transcriptome analysis
Source: PLoS One. 2018 Dec 12;13(12):e0208771. doi: 10.1371/journal.pone.0208771 (PMC6291152; doi:10.1371/journal.pone.0208771)
Supplement: S1 Table — (DOC) [file pone.0208771.s001.doc]

**S1 Table. Primer sequence of genes**

| Gene | Upstream Primer | Downstream Primer |
| --- | --- | --- |
| MSTRG.15647 | TCACTCATCTGCCTCACATTC | TTCCCGTAGCCACAAGC |
| MSTRG.33946 | TAGCATTGTTTGTTTCGTGTTG | TCCCGTCGCCATTGTCT |
| MSTRG.46080 | AGAGCCACCTTGTCATTTC | TCTATGCAACCCAACCTTA |
| MSTRG.12329 | TGCGTAACCCAGTTGTAAA | CAGCTCGTTCCTCCGATA |
| MSTRG.15171 | TCGGCGACTCTACAACTGAA | AAGAGGCGTATGCTCGATTT |
| MSTRG.26332 | TCATCCTACGTGGCGATACCT | GCTTCGTGGGTCGTTTCTG |
| MSTRG.26097 | CACCACCGCCTCATCATCC | TCGCTATCGCCATCTTGCTC |
| MSTRG.21401 | GCACATTCGCAGCTCAAAC | CATGTCCAAGGCCGATGAC |
| MSTRG.52145 | CTTATCTGTTTGAGCTGGAGTAGTTG | GGGCTGCGAAATGAAAGAAT |
| MSTRG.54748 | CCCCTCCTCTGAACGAA | ATGGGCAGCAGTTTTCTC |
| MSTRG.12960 | TCCCAACCATTGACCTTTC | TCGCCCAGATCACCATTAA |
| MSTRG.14947 | CATGACTTTGTGCAGGCTTAG | GCTCTTATTGGAGTGGGTGTAT |
| *LcGA2ox1* | ATGGTGGTTCTGTCACAGCCAG | TCAATGGGCAGCAGTTTTCTC |
| *LcGA2ox2* | ATGGTTTCTCCTTCTGTTCTAC | TCACGACGCAATTTTGTTGCC |
| *LcGA2ox3* | ATGGACTCGGACCCACCATTT | TTAATGGATTCTTAGAAGAAATC |
| *35S::LcGA2ox1* | GGACTCTAGAGGATCCATGGTGGTTCTGTCACAGCCAG | GATCGGGGAAATTCGAGCTCTCAATGGGCAGCAGTTTTCTC |
| *35S::LcGA2ox2* | GGACTCTAGAGGATCCATGGTTTCTCCTTCTGTTCTAC | GATCGGGGAAATTCGAGCTCTCACGACGCAATTTTGTTGCC |
| *35S::LcGA2ox3* | GGACTCTAGAGGATCCATGGACTCGGACCCACCA | GATCGGGGAAATTCGAGCTCTTAATGGATTCTTAGAAGAAATC |
