# Supplementary figures and images for: Differential gene expression between the vigorous and dwarf litchi cultivars based on RNA-Seq transcriptome analysis
Source: PLoS One. 2018 Dec 12;13(12):e0208771. doi: 10.1371/journal.pone.0208771 (PMC6291152; doi:10.1371/journal.pone.0208771)

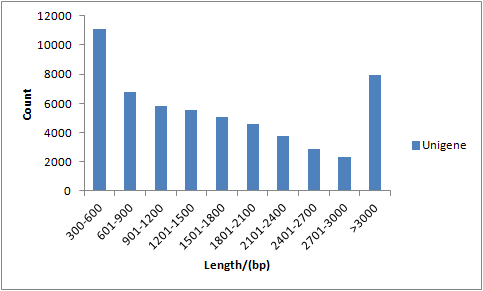

Supplement: S1 Fig — The x-axis denoted the length range of all groups. The y-axis denoted the number of unigenes in each group. (TIF) [file pone.0208771.s004.tif]

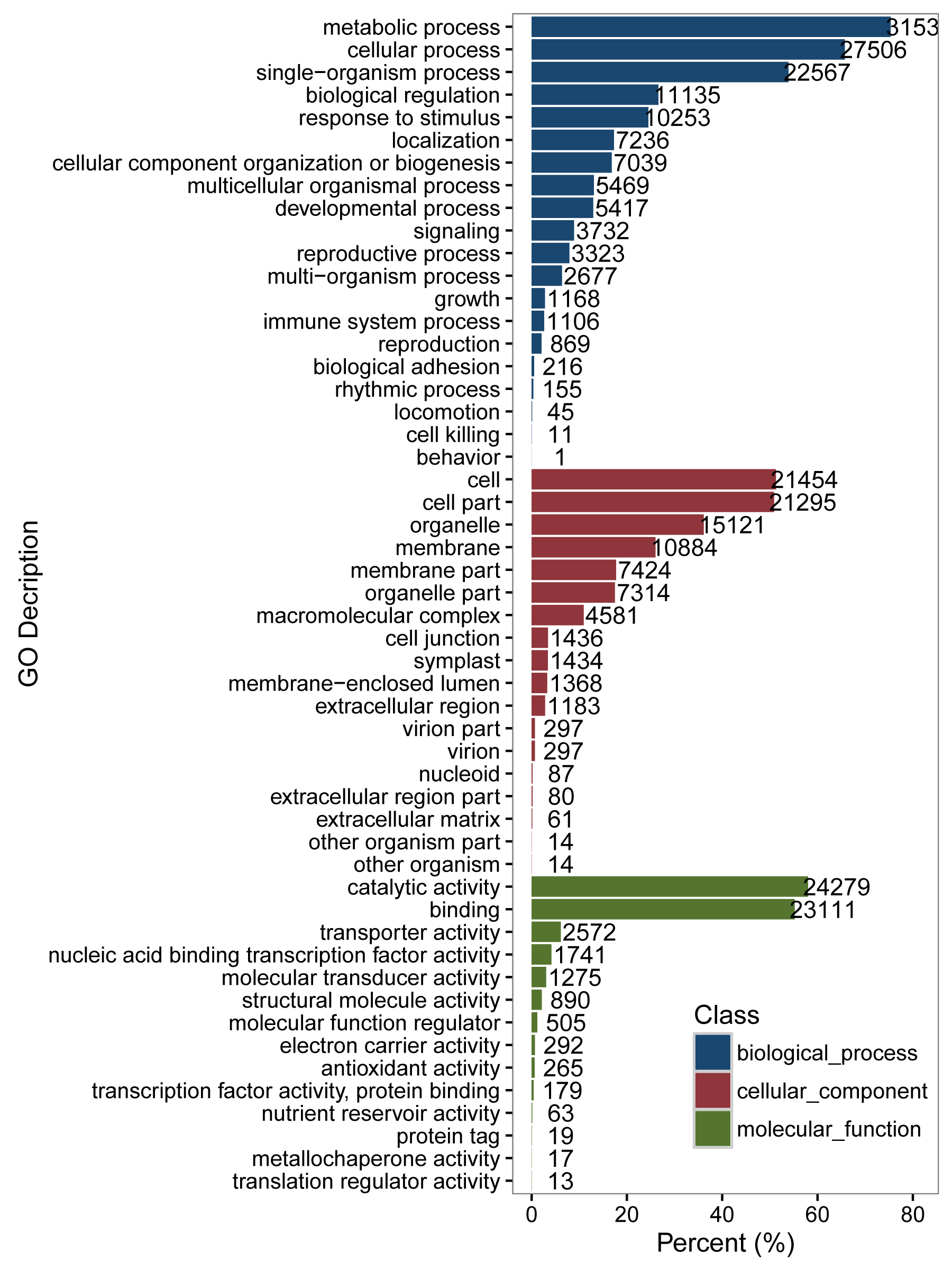

Supplement: S2 Fig — The number of gene GO terms in each functional subcategory was presented as the percentage of GO terms for that subcategory out of the total GO terms. (TIF) [file pone.0208771.s005.tif]

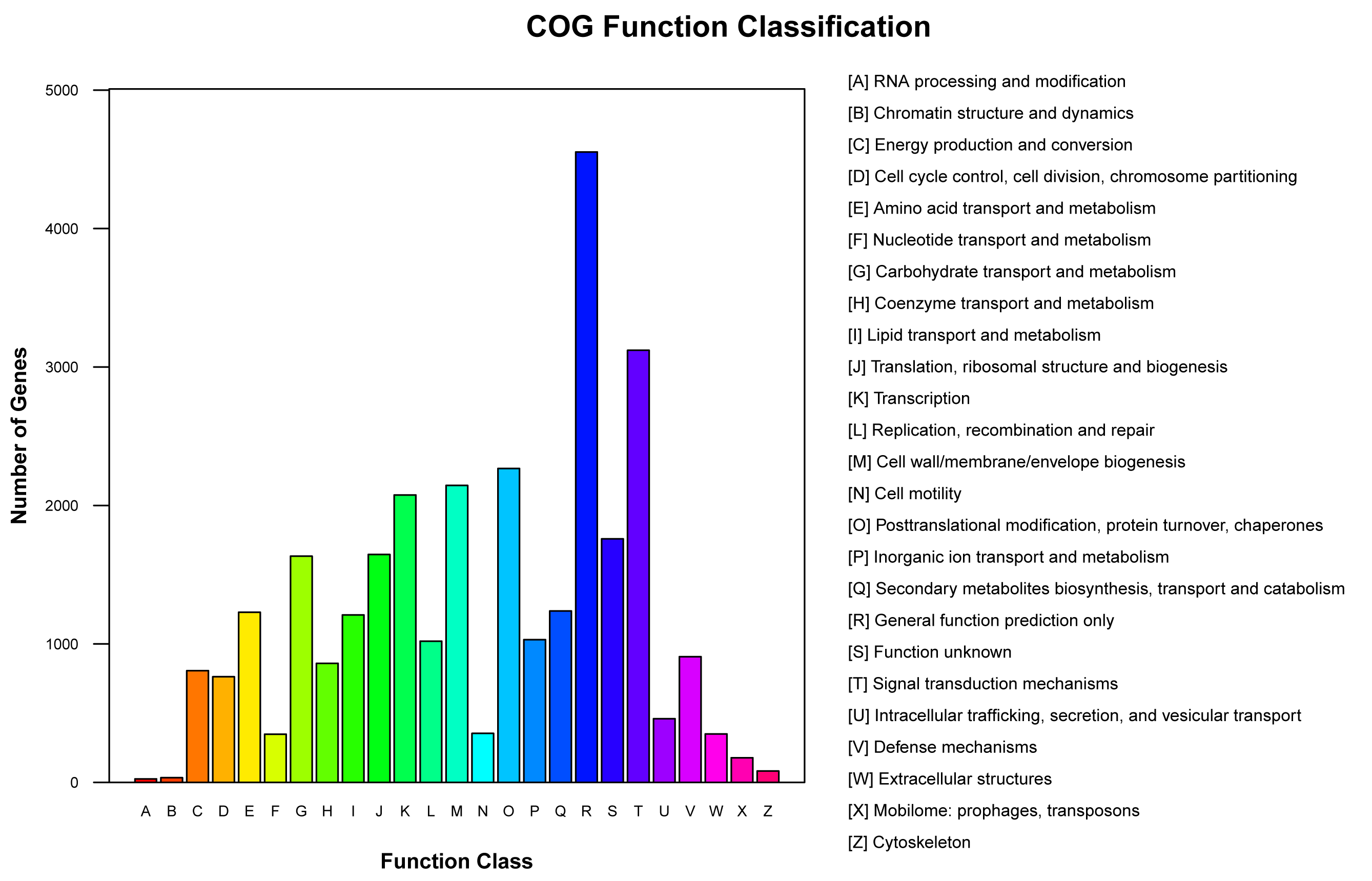

Supplement: S3 Fig — The y-axis denoted the number of unigenes in each group. The x-axis denoted the functional description of each group. Details were shown in the right part of the graph. (TIF) [file pone.0208771.s006.tif]

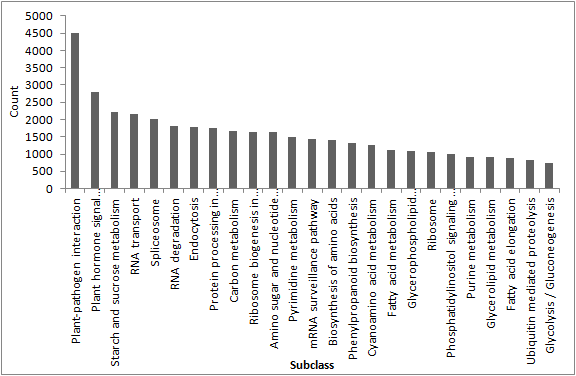

Supplement: S4 Fig — The y-axis denoted the number of unigenes in each group. The x-axis denoted subclass of KEGG. (TIF) [file pone.0208771.s007.tif]

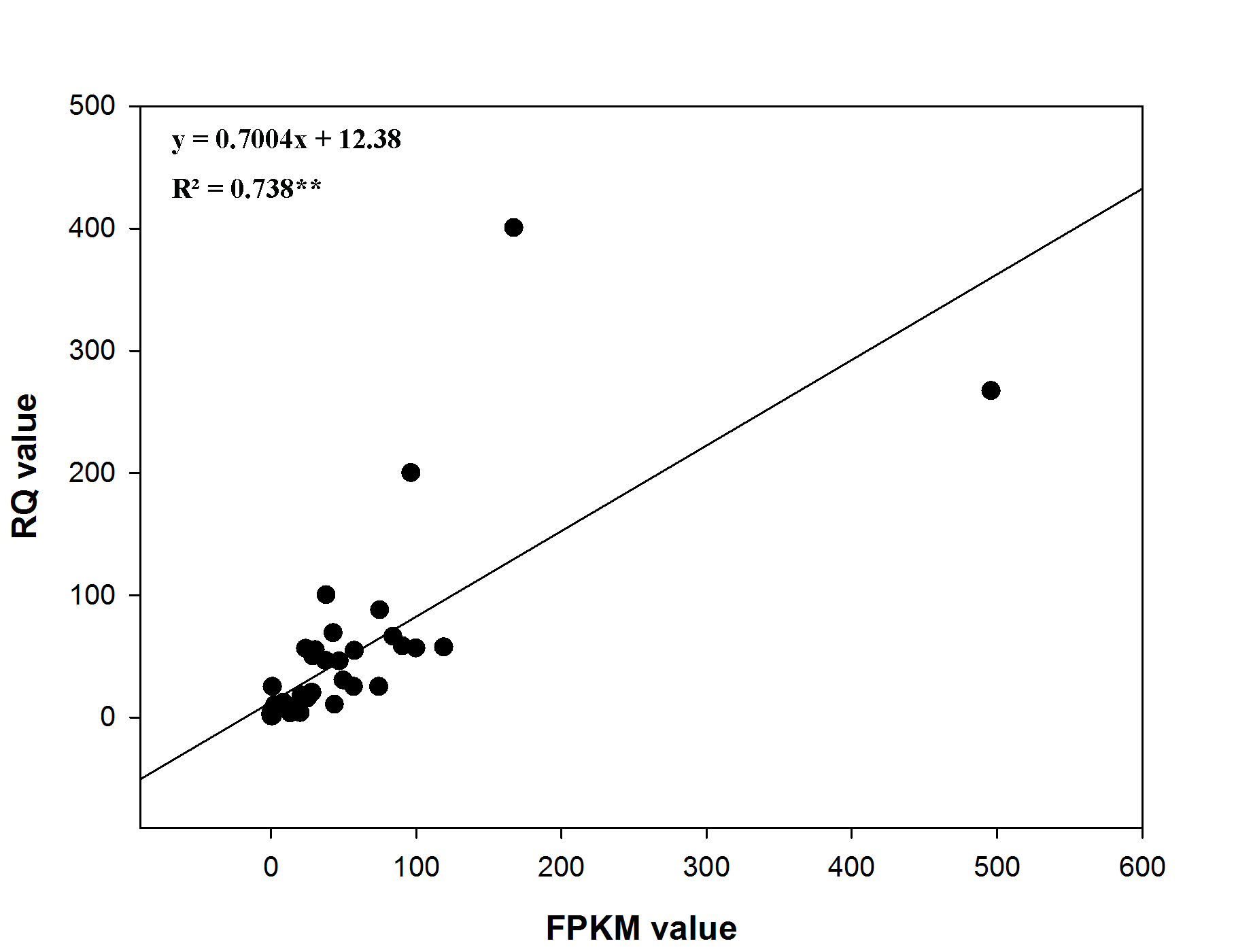

Supplement: S5 Fig — The real-time PCR log2 values (x-axis) were plotted against colorationstages (y-axis). **indicates a significant difference at p ≤ 0.01. (TIF) [file pone.0208771.s008.TIF]
